# A novel hemostatic suture technique for uterine rupture complicated by placenta accreta spectrum: A case report

**DOI:** 10.1097/MD.0000000000050441

**Published:** 2026-09-04

**Authors:** Ruiyan Shang, Jing Yang, Li Li, Yiyi Zhuge

**Affiliations:** a Department of Gynecology, Hangzhou Women’ s Hospital, Hangzhou, China; b Department of Obstetrics and Gynecology, Peking University Third Hospital, Beijing, China; c Department of Obstetrics and Gynecology, Zhengzhou Central Hospital Affiliated to Zhengzhou University, Zhengzhou, China.

**Keywords:** case report, innovative surgical technique, obstetric emergency, obstetric hemorrhage, placenta accreta spectrum, surgical hemostasis, uterine rupture

## Abstract

**Rationale:**

Uterine rupture secondary to the placenta accreta spectrum (PAS) is a life-threatening obstetric emergency. Intractable massive hemorrhage in this setting often necessitates emergent hysterectomy, while effective fertility-sparing surgical options for such critical bleeding remain limited. This report highlights the utility of a novel suturing technique for achieving effective hemostasis and fertility preservation in patients with PAS-related uterine rupture.

**Patient concerns:**

A 36-year-old Asian woman diagnosed with PAS presented with spontaneous uterine rupture at 16+ weeks of gestation, complicated by severe refractory intra-abdominal hemorrhage.

**Diagnoses:**

The diagnosis of PAS-related uterine rupture was confirmed based on the patient’s previous cesarean delivery history, clinical manifestations, preoperative imaging findings, and postoperative pathological results.

**Interventions:**

A novel technique combining anteroposterior traction and bilateral reinforcement sutures of the lower uterine segment was applied for hemostasis, with an estimated intraoperative blood loss of 400 mL.

**Outcomes:**

The patient recovered uneventfully and was discharged on postoperative day (POD) 11; regular menses persisted at 1-year follow-up.

**Lessons:**

PAS-related uterine rupture is easily misdiagnosed or delayed owing to variable clinical presentations. Conventional hemostatic approaches are often ineffective for refractory severe hemorrhage. This novel combined suturing technique offers a simple, feasible fertility-sparing strategy to avoid unnecessary hysterectomy and improve maternal outcomes in catastrophic obstetric bleeding.

## 1. Introduction

The placenta accreta spectrum (PAS) is a complex obstetric condition defined by abnormal placental invasion into the myometrium. It is histologically classified into placenta accreta, increta, and percreta based on the depth of invasion.^[1]^ Over the past decade, the global incidence of PAS has risen dramatically, closely following the increase in cesarean delivery rates.^[2]^ According to current estimation, the incidence rate of PAS may increase to 1 in 200 women undergoing cesarean delivery by 2025,^[3]^ posing a significant challenge in modern obstetrics.

Uterine rupture secondary to placenta percreta is a rare but catastrophic complication within the PAS. Uterine rupture can lead to disseminated intravascular coagulation, which requires massive transfusion and intensive care. Clinicians may resort to hysterectomy due to uncontrollable bleeding.^[4]^ Recent studies report a maternal mortality rate of up to 7%^[5]^ and an increased risk of neonatal morbidity.^[6]^ Diagnosing this condition is challenging due to its varied symptoms, ranging from acute abdominal pain with hemodynamic instability to subtle, nonspecific signs that often lead to delays.^[7,8]^ Management of PAS has seen a significant shift in recent years. While cesarean hysterectomy was historically standard for massive hemorrhage,^[9]^ the updated FIGO consensus guidelines now strongly advocate uterine-preserving strategies when clinically appropriate.^[10]^ This reflects the importance of fertility preservation and the psychological impact of hysterectomy. Current management includes techniques like the “Triple P” procedure, uterine artery embolization, and various surgical compression sutures, have emerged as viable alternatives to preserve fertility, providing the opportunity for future pregnancies.^[11,12]^ However, existing surgical techniques have limitations. For instance, the B-Lynch suture and its modifications, though effective for hemorrhage from the uterine body, are notably less effective for controlling bleeding from the lower uterine segment and cervix, which is common in PAS cases.^[13]^ Various specialized folding sutures were developed to control bleeding in this anatomical region, such as circular isthmic cervical compression sutures,^[14]^ modified suture technique,^[15]^ and cervical lifting suture, designed specifically for lower segment hemorrhage.^[16]^

Despite these advancements, managing hemorrhage from the lower uterine segment, especially in cases of uterine rupture, remains challenging. This case report describes successful hemostasis achieved with a novel combined suturing technique – anteroposterior traction sutures plus bilateral lower uterine segment reinforcement sutures – to manage life-threatening massive hemorrhage secondary to second-trimester uterine rupture complicated by placenta percreta. This innovative suturing technique provided an easy and efficient surgical choice, and a technically feasible fertility-preserving strategy for managing catastrophic hemorrhage in PAS-related uterine rupture. It also discusses the technique’s clinical promotion value.

## 2. Case presentation

A 36-year-old Asian woman (G4P2) presented to the emergency department overnight with a 16+-week gestation and half a day of nausea and vomiting. The patient developed intermittent lower abdominal discomfort after eating rice dumplings and cured meat half a day before admission, accompanied by transient abdominal pain and 3 episodes of emesis. Vomitus contained only gastric contents. Transabdominal ultrasound demonstrated a fetal heart rate of 152 beats per minute (bpm), fetal gastroschisis, and an absence of pelvic free fluid. Initial diagnoses included acute gastroenteritis, fetal gastroschisis, mid-trimester gestation (G4P2, 16+ weeks), and previous uterine cesarean scar.

The patient had 2 prior term lower-segment cesarean deliveries in 2011 and 2019. At the 7-week prenatal ultrasound for this pregnancy, the inferior margin of the gestational sac was measured 9 mm away from the cesarean scar. A follow-up ultrasound performed at 14 weeks confirmed placental attachment to the left anterior uterine wall with complete coverage of the internal cervical os.

### 2.1. Physical examination upon admission

Temperature, 36.9°C; pulse, 98 beats per minute (bpm); blood pressure (BP), 135/85 mm Hg (1 mm Hg = 0.133 kPa); oxygen saturation, 98%. The abdomen was soft, without tenderness or rebound tenderness, and no uterine contractions were palpable. Fetal heart rate was 150 bpm.

### 2.2. Laboratory results

Complete blood count: white blood cell 30.9 × 10^9^/L, neutrophil 89.3%, hemoglobin 91 g/L, hematocrit 0.264, platelet 264 × 10^9^/L.
Biochemistry: total protein 69.5 g/L, albumin 36.8 g/L, C-reactive protein 32.17 mg/L; hepatic enzymes and bilirubin normal.
Coagulation: prothrombin time 11.3s, thrombin time 15.0s, activated partial thromboplastin time 19.3s, d-Dimer 23,430 μg/L.

### 2.3. Post-admission management and clinical follow-up

Following admission, the patient received intravenous antibiotics, with subsequent improvement in abdominal discomfort, nausea, and emesis.

### 2.4. Morning laboratory results on the second hospital day

Complete blood count: white blood cell 16.8 × 10^9^/L, neutrophil 80.3%, hemoglobin 62 g/L, hematocrit 0.18, platelet 191 × 10^9^/L.
Biochemistry: total protein 56.2 g/L, albumin 30.4 g/L.
Coagulation: prothrombin time 11.9 s, activated partial thromboplastin time 21.2 s, thrombin time 14.5 s, D-Dimer 23,970 μg/L.

### 2.5. Subsequent clinical course

**Figure 1.**
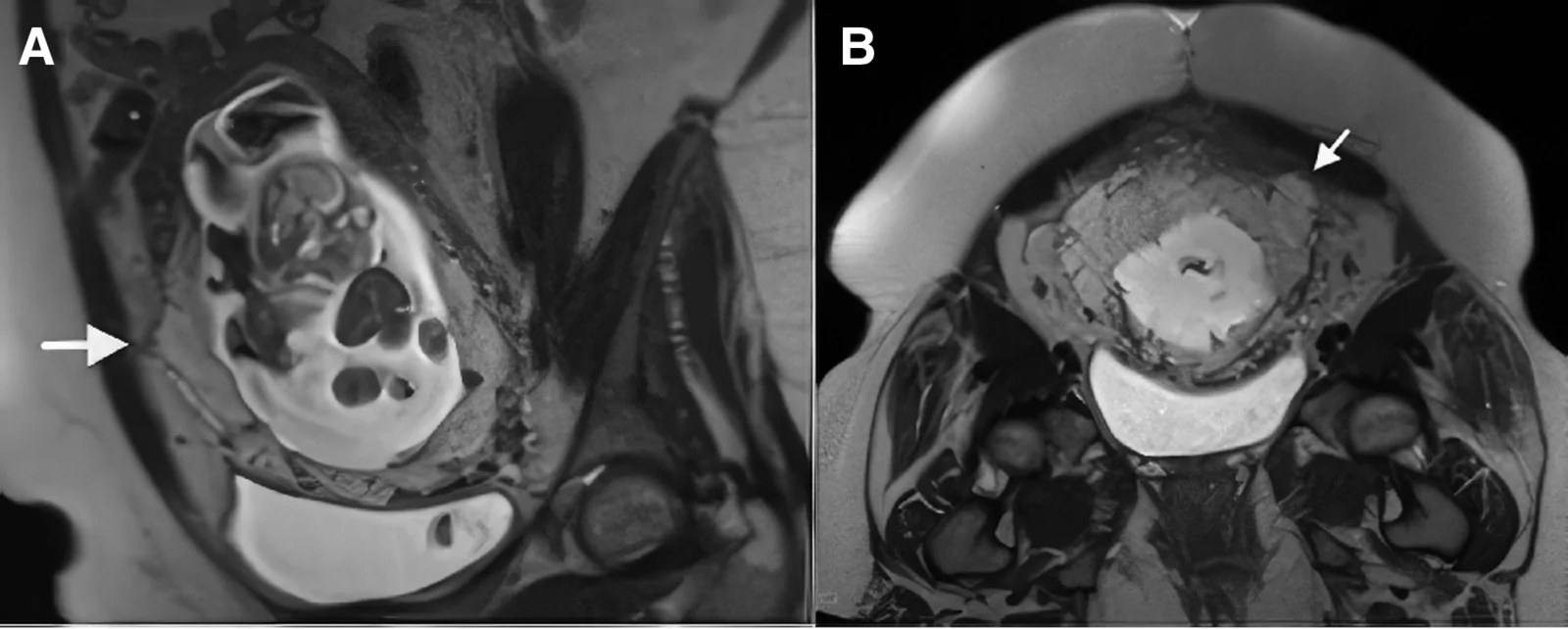
MRI demonstrates placental invasion into the myometrium with external protrusion. (A) Sagittal view of the uterus showing placental implantation. (B) Transverse view of the lower uterine segment. MRI = magnetic resonance imaging.

Mild lower abdominal pain recurred in the patient at roughly 15:00 on the same day. Physical examination elicited mild lower abdominal tenderness, with no detectable uterine contractions. An emergency repeat transabdominal ultrasound demonstrated free intraperitoneal fluid with depths measured as follows: 3.3 cm within the hepatorenal recess, 0.8 cm in the splenorenal recess, and 2.6 cm in the left lower peritoneal cavity. Intraperitoneal hemorrhage was highly suspected, with placental-origin bleeding regarded as the leading diagnosis. After thorough counseling with the patient concerning all associated risks and anticipated clinical outcomes, she opted for pregnancy termination and strongly requested fertility-sparing surgery. All requisite preoperative workups were completed expeditiously to facilitate emergency operative management. Pelvic magnetic resonance imaging (MRI) and contrast-enhanced whole-abdominal computed tomography (CT) demonstrated multifocal intraperitoneal hemorrhage, central placenta previa, extensive placental implantation within the lower uterine segment, signs of uterine wall penetration along the left anterior myometrium, and suspected uterine rupture (Fig. 1).

### 2.6. Differential diagnosis

Initial differential diagnoses included threatened abortion, acute gastroenteritis, or hemorrhage from a non-gynecological abdominal organ. The patient’s history of prior cesarean sections and the earlier ultrasound finding of a low-lying gestational sac heightened the suspicion for PAS, which was ultimately confirmed intraoperatively.

### 2.7. Treatment and prognosis

A multidisciplinary discussion was held preoperatively by gynecology, obstetrics, anesthesiology, and intensive care medicine. The multidisciplinary team (MDT) reached a consensus to perform an exploratory laparotomy with cesarean extraction of the fetus, fully cognizant of the elevated risk of hemorrhage and the potential necessity for a hysterectomy.

**Figure 2.**
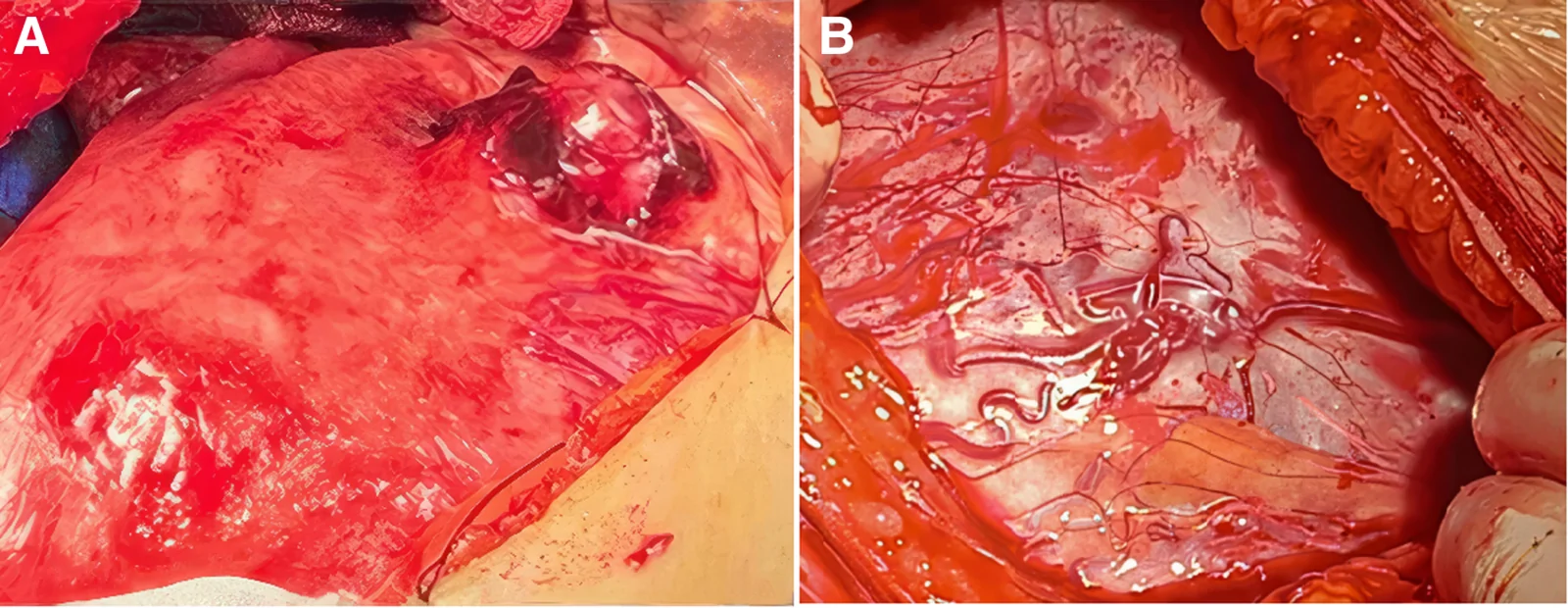
The rupture, located at the anterior lower uterine wall, was encircled by a dilated tortuous vascular plexus derived from placenta percreta.

Intraoperative exploration revealed massive pelvic and abdominal hemoperitoneum with clots, and estimated intraoperative blood loss was 1600 mL. Severe vesicouterine adhesions were present, and the uterine body had good blood supply. Placenta percreta (2 × 3 cm) was located at the left lower uterine segment. The serosal layer was absent, and active bleeding was present. Additionally, rich neovascularization was observed in the surrounding area (Fig. 2).

**Figure 3.**
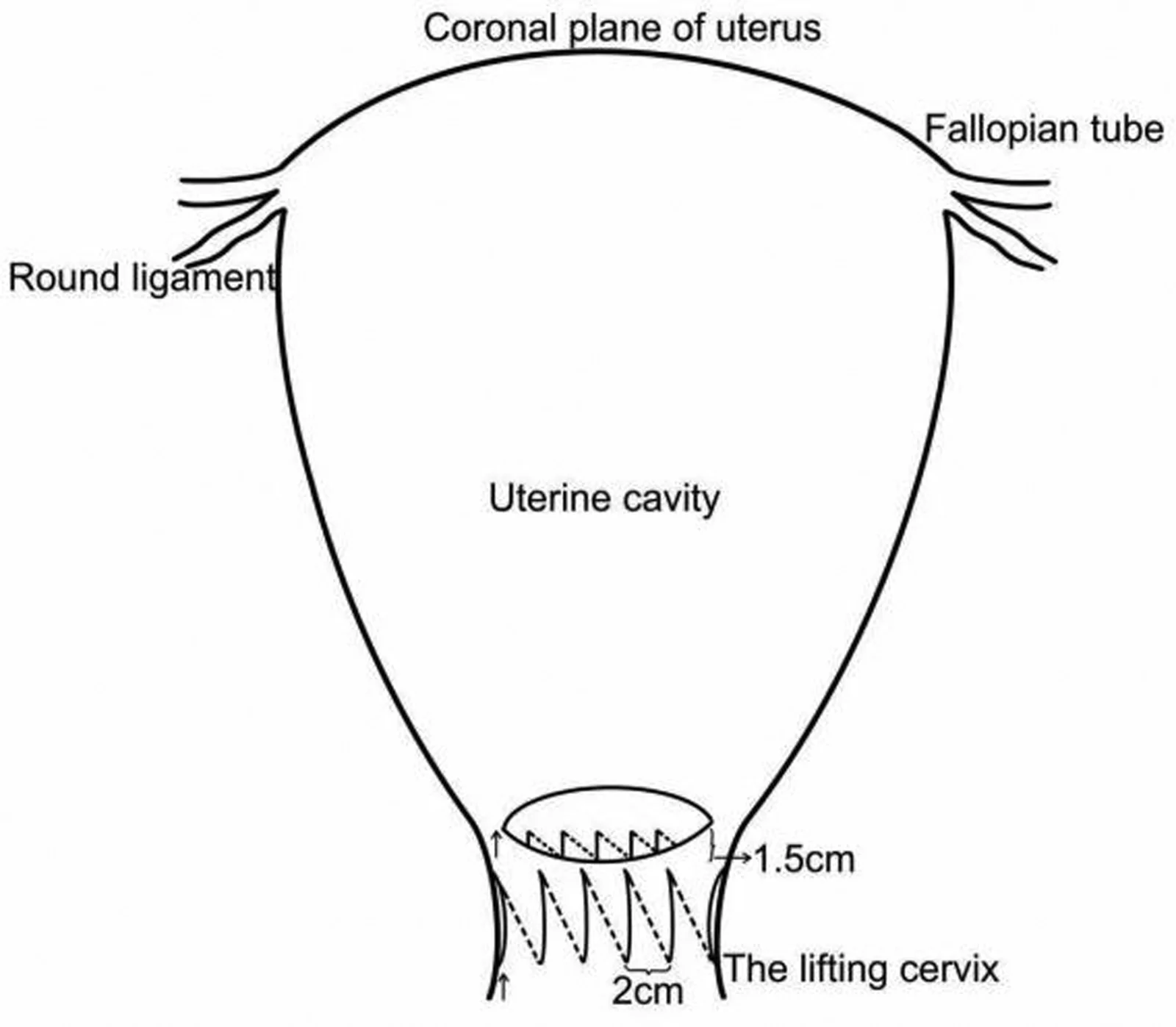
Anteroposterior traction suture technique of the lower uterine segment.

**Figure 4.**
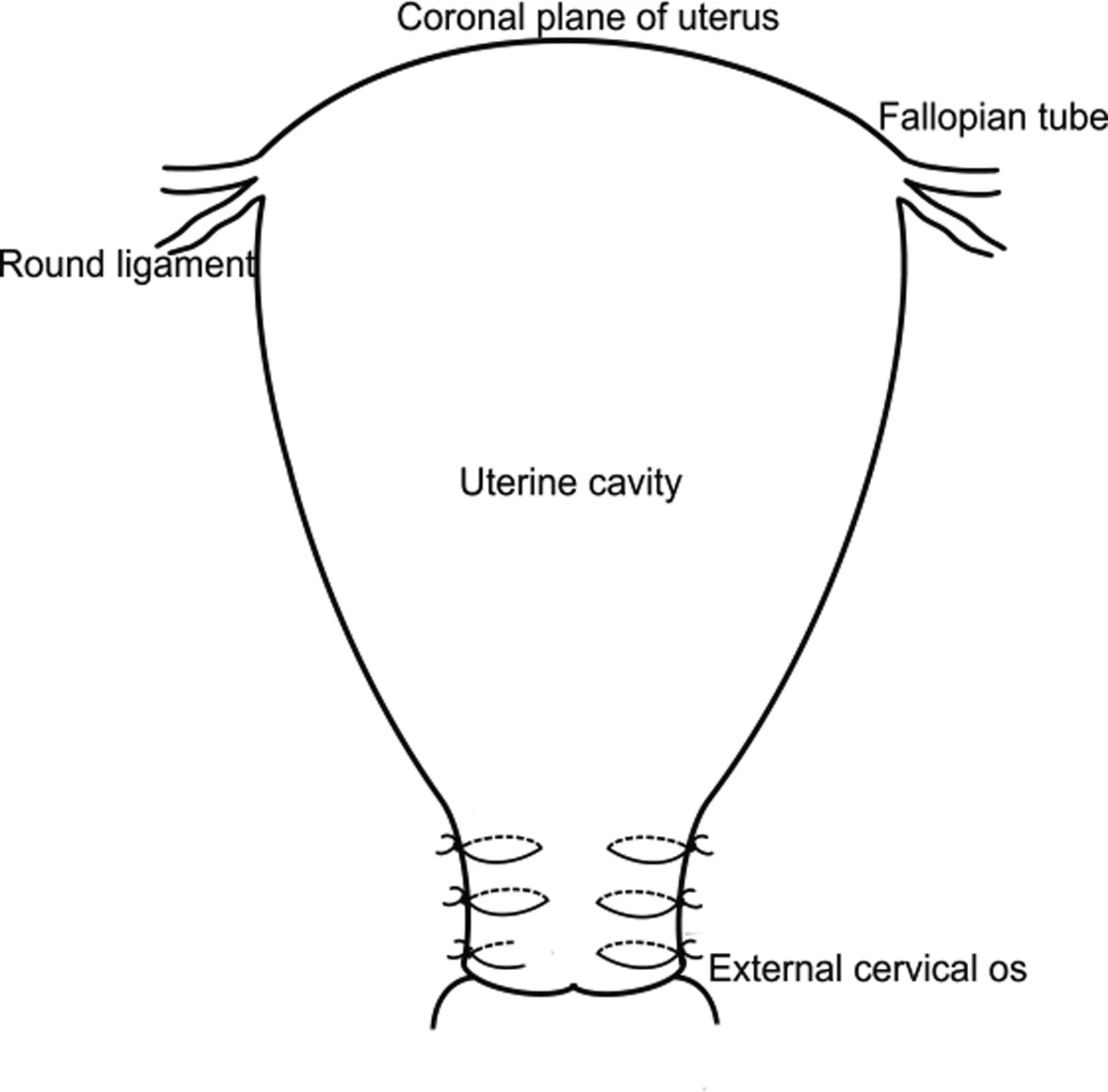
Bilateral reinforcing suture technique of the lower uterine segment.

The vesicouterine peritoneum was incised, followed by sharp dissection and mobilization of the bladder down to the external cervical os. Iatrogenic bladder rupture occurred during dissection, which directly exposed placental invasion into the bladder wall. A cervical tourniquet was placed at the internal os for devascularization of the cervix and pericervical vessels. A longitudinal hysterotomy was performed to deliver a stillborn fetus complicated by an abdominal wall defect (evisceration of the liver, bowel, and stomach) and right foot syndactyly. After fetal extraction, circumferential placental adherence was identified over the anterior, posterior, and left uterine walls, fully occluding the internal cervical os. The placental infiltrated uterine tissue was resected, and residual placental tissue was cleared via clamp dissection and blunt stripping. The hysterotomy was reconstructed into an inverted T incision. Severe hemorrhage arose from the thin, atonic lower uterine segment. Hemostasis attempts, including intramyometrial carboprost tromethamine (250 μg) and routine suturing, failed to achieve bleeding control. A novel combined hemostatic suture was applied with 1-0 absorbable sutures, combining anteroposterior traction sutures and bilateral lower uterine segment reinforcing sutures to achieve hemostasis (Figs. 3 and 4). Intrauterine packing was added for adjunct hemostasis, which finally achieved effective bleeding control. The total estimated blood loss reached 2000 mL, requiring transfusion of 7 units packed red blood cells and 390 mL fresh frozen plasma. Intraoperative vital signs were stable. The patient was admitted to the intensive care unit (ICU) for intensive postoperative monitoring and then transferred to the general ward.

Piperacillin-tazobactam was given intravenously for perioperative antibiotic prophylaxis. The intrauterine pack was removed at 48 hours postoperatively with scant vaginal bleeding. The pelvic drain was removed on POD 5, and the urinary catheter was discontinued on POD 10. The patient recovered uneventfully and was discharged on POD 11. No adverse symptoms were noted at 4-week and 3-month follow-ups, and contraception was strongly recommended. Normal menstruation was confirmed at 6-month and 1-year follow-up visits.

### 2.8. Surgical technique

Anteroposterior traction suture technique of the lower uterine segment: The cervical internal os was retracted superiorly. A 1-0 absorbable suture was introduced horizontally, 1 cm inferior to the cervical internal os at the left lateral margin of the uterine incision, exiting 1.5 cm below the inferior edge. The suture was then run continuously under traction to the right lateral margin, spacing the suture lines approximately 2 cm apart. This creates circumferential compression and reinforcement of the lower segment (Fig. 3).

Bilateral reinforcing suture technique of the lower uterine segment: Horizontal 1-0 absorbable sutures were placed at the level of the cervical external os, positioned 0.5–1 cm medial to the lateral uterine margins. Subsequently, 2–4 vertical interrupted sutures were applied medial to these, based on hemodynamic rationale to reduce vascular perfusion to the bleeding lower segment (Fig. 4).

## 3. Discussion

This case highlights the substantial clinical challenges posed by second-trimester uterine rupture secondary to PAS. It also demonstrates the successful application of a novel uterus-preserving surgical technique for the management of this life-threatening condition. The patient initially presented with upper abdominal pain triggered by food ingestion, which was initially misdiagnosed as acute gastroenteritis. This diagnostic pitfall is consistent with commonly reported clinical dilemmas in the existing literature.^[17]^ Atypical clinical manifestations of PAS-related uterine rupture often lead to delayed and suboptimal clinical intervention. A recent systematic review further confirms that nonspecific abdominal pain and vaginal bleeding constitute the most prevalent initial symptoms of second-trimester uterine rupture secondary to PAS. Such vague presentations render the condition highly prone to misdiagnosis, thereby underscoring that a high clinical suspicion index is essential for timely and accurate diagnosis.^[18]^

Achieving adequate hemostasis during surgery for PAS disorders poses substantial clinical challenges. Patients with a history of multiple cesarean deliveries frequently exhibit extensive pelvic adhesions, a thin, highly vascularized lower uterine segment, a bulky implanted placenta, and profound pelvic neovascularization. In cases complicated by placenta percreta, invasive placental tissue may additionally infiltrate adjacent structures, including the urinary bladder, intestines, cervix, and parametrium. The core strength of our novel suturing technique is its targeted hemostatic effect on the thin, poorly contractile lower uterine segment – an anatomical defect caused by repeated cesarean deliveries and invasive placental implantation. By contrast, classic compressive suturing methods such as the B-Lynch procedure are primarily designed to manage uterine atony confined to the uterine corpus.^[13]^ Our novel suturing technique achieves robust hemostasis via 2 core operative steps. First, anteroposterior traction sutures lift the lower uterine segment and deliver circumferential compression through a ligation effect, mechanically occluding blood flow from the descending uterine artery branches and cervical vascular plexus. Second, bilateral reinforcing sutures further diminish the overall blood perfusion of the lower uterine segment while providing structural support to the eroded, attenuated myometrium – an effect unattainable with conventional single-point sutures or intrauterine packing.^[19]^ Furthermore, this suturing approach carries substantial clinical translational value. First, it imposes minimal demands on specialized equipment; the entire procedure can be performed using routine absorbable sutures, eliminating reliance on costly vascular closure devices or tissue sealants. Second, the technique features a gentle learning curve and places relatively low technical burdens on surgeons. For primary hospitals lacking interventional radiology facilities or managing patients with hemodynamic instability precluding interventional procedures, mastery of this method can secure critical time for life- and uterus-preserving salvage. This stands in stark contrast to interventions including the Triple-P procedure and internal iliac artery balloon occlusion, whose execution relies heavily on the sophisticated infrastructure and resources available only at tertiary medical centers.^[20,21]^

The mechanistic rationale underlying our technique aligns with 2 recently reported hemostatic strategies: multi-square suturing for targeted placental bed compression^[22]^ and cervical elevation sutures designed exclusively for lower uterine segment hemorrhage control.^[23]^ Nevertheless, our integrated dual-suture approach combines traction elevation and myometrial reinforcement to deliver a more holistic hemostatic solution. Notably, surgical intervention was administered more than 20 hours following uterine rupture in this complicated case; the favorable clinical outcome observed herein validates that this suturing technique can achieve satisfactory hemostasis even under suboptimal emergent conditions.

This further reinforces the emerging consensus that individualized, multimodal surgical approaches constitute the cornerstone of fertility-sparing management for complicated PAS. A published systematic review underscores that successful hemostasis hinges on surgeons’ flexible deployment of diverse hemostatic techniques tailored to each patient’s distinct bleeding location and uterine anatomical status.^[24]^

Several limitations of the present case report warrant Acknowledgments. First, the single-case design restricts the generalizability of our findings; large-scale prospective cohorts are required to further validate the efficacy of this combined suturing technique. Second, long-term clinical outcomes remain unevaluated, including menstrual recovery, the incidence of intrauterine adhesions, and gestational risks in subsequent pregnancies such as recurrent uterine rupture and recurrent PAS. Extended standardized follow-up and thorough preconception counseling are therefore essential for this patient population. Recent literature addressing fertility outcomes following fertility-sparing management for PAS underscores that all patients should be fully counseled regarding the substantial hazards of subsequent gestation, alongside the mandatory requirement for rigorous preconception evaluation and close antenatal monitoring.^[25]^ Furthermore, this suturing procedure ought to be administered by surgeons proficient in complex obstetric surgery within a multidisciplinary collaborative framework, with emergency hysterectomy retained as a definitive life-saving rescue strategy at all times.

## 4. Conclusion

The combined technique of lower uterine segment lifting suture and bilateral uterine wall reinforcement suture represents a novel, technically feasible, and effective strategy for managing life-threatening hemorrhage caused by PAS-related uterine rupture, particularly in patients who wish to preserve fertility. This surgical approach provides a reliable fertility-sparing alternative for appropriately selected and counseled individuals. While this case report offers promising preliminary evidence, further large-scale and collaborative studies are warranted to validate its safety, efficacy, and long-term clinical outcomes.

## Author contributions

**Conceptualization:** Ruiyan Shang, Jing Yang, Yiyi Zhuge.

**Data curation:** Ruiyan Shang, Yiyi Zhuge.

**Supervision:** Ruiyan Shang, Jing Yang, Li Li, Yiyi Zhuge.

**Writing – original draft:** Ruiyan Shang.

**Writing – review & editing:** Ruiyan Shang, Jing Yang, Li Li, Yiyi Zhuge.
